# Do No Harm: The Urgent Need to Reform Short-Term Global Health Experiences

**DOI:** 10.5334/aogh.2525

**Published:** 2019-06-17

**Authors:** Keith Martin

**Affiliations:** 1 Consortium of Universities for Global Health, US

## Abstract

This is a review of an article by Virginia Rowthorn et al. that will be published in AOGH.

Largely unregulated, short-term experiences in global health (STEGHs) often venture between the unethical and the illegal. In “Not Above the Law: A Legal and Ethical Analysis of Short-term Global Health Experiences,” Virginia Rowthorn et al. shine a bright light on these ignored and often dangerous programs. Well-meaning but unqualified individuals from high-income countries are engaging in clinical activities that they have no business doing in low-income countries (LICs). These are massively under-resourced environments with overwhelming patient loads, many of whom have complex health needs.

This situation has developed due to a toxic mix of economic, religious, and historical forces that have combined to exploit a genuine desire by many people, especially the young, to serve and relieve the suffering of those in need. The organizations offering these programs are also capitalizing on those seeking a competitive advantage in gaining entrance into a professional school such as medical or nursing.

The authors vividly describe grossly inappropriate activities by undergraduates, overzealous medical students and even high school students participating in clinical activities where they have no right to be. Many of these activities would be illegal in the United States.

The global health experiences in the article are referring to those undertaken in clinical environments. They did not include nonclinical STEGHs that could safely enable students, with proper supervision and agreement from the host community, to experience the social determinants of health, for example, education, housing, environmental issues, water, and sanitation. Such programs would expose students to many pressing global health challenges, other cultures, and working and living in low-resource environments while minimizing the risks to patients and participants.

So, how can we right this injustice and reform STEGHs?

First, change should be based on the following principles: do no harm, mutual respect, equity, they do not drain the host’s assets, reciprocity, effective communication, and ensuring that the majority of the benefits from the collaboration accrue to the LIC institution.

Based on these principles STEGH reform should include:

1. Banning all clinical STEGHs undertaken by high school students, undergraduates, and unlicensed individuals.
2. An obligation that high-income institutions oversee their programs and be responsible to the host LIC institution for any errors committed by their students.
3. Prohibiting students from engaging in any activities overseas that they are not qualified to perform in their home country.
4. Memoranda of Understanding should be created between the high-income and host institution outlining each partner’s responsibilities, activities, and benefits.
5. Benefits to the LIC should be clearly outlined, long-term, sustainable, and congruent with their needs. These could include access to trainers, curricula, and research libraries online to strengthen education and research capabilities. STEGHs provide an entry point to build collaborative, long-term, impactful capacity building in LICs to address their growing deficits in healthcare workers and management capabilities.
6. Bidirectional learning opportunities should be made available to LICs by providing free online training.
7. Quality pre-deployment and post-deployment training should be obligatory for all participants.
8. Prohibit nonmedical STEGH participants from participating or observing any clinical services. This is a matter of patient safety, respect, and dignity.
9. Participants in STEGHs must adhere to local laws and licensing requirements.
10. Accreditation bodies in the United States should develop rules governing STEGHs, assess their members’ compliance with these rules, and penalize those that violate them.

The paper’s authors also address the issue of drug donation and exportation. They outline four core principles that form the basis of good medicine donation practices. What was not addressed were medical devices. Low-income countries have become a dumping ground for them. These donations are rooted in a desire to address the enormous equipment needs in LICs. However, the donated equipment often cannot be maintained, fails to be interoperable, or is not relevant to the setting to where they were sent.

Professional licensing bodies should work with the private sector and define a narrow scope for effective medical equipment donations. This would begin to reduce the sea of broken or inappropriate donated medical equipment that burden many LIC health institutions.

“Not Above the Law” outlines a powerful case to urgently reform STEGHs. It is a call to action for the Justice Departments, academia, professional institutions and accreditation bodies in high income nations to shut down practices that would be illegal if undertaken within their nations. It is a chance to protect the dignity and lives of those who may lack the agency and options to access quality care when they are ill and prevent well-meaning people from inadvertently harming themselves or others. Institutions in high-income nations have a duty to act. The responsibility rests on our shoulders.

